# A Pilot Study on the Analysis of Circulating miRNA Upregulation in Laryngeal Cancer

**DOI:** 10.3390/diseases13040101

**Published:** 2025-03-30

**Authors:** Crina Oana Pintea, Marius Pricop, Edward Seclaman, Nicolae Constantin Balica, Kristine Guran, Delia Ioana Horhat, Cristian Ion Mot

**Affiliations:** 1 Doctoral School, “Victor Babes” University of Medicine and Pharmacy Timisoara, Eftimie Murgu Square 2, 300041 Timisoara, Romania; crina.pintea@umft.ro (C.O.P.); guran.kristine@umft.ro (K.G.); 2 Department IX, Discipline of Otolaryngology, “Victor Babes” University of Medicine and Pharmacy Timisoara, Eftimie Murgu Square 2, 300041 Timisoara, Romania; balica@umft.ro (N.C.B.); horhat.ioana@umft.ro (D.I.H.); ion.mot@umft.ro (C.I.M.); 3 Discipline of Oral and Maxillo-Facial Surgery, Faculty of Dental Medicine, “Victor Babes” University of Medicine and Pharmacy Timisoara, Eftimie Murgu Square 2, 300041 Timisoara, Romania; 4 Department of Biochemistry and Pharmacology, “Victor Babes” University of Medicine and Pharmacy Timisoara, Eftimie Murgu Square 2, 300041 Timisoara, Romania; eseclaman@umft.ro; 5 Center for Complex Networks Science, “Victor Babes” University of Medicine and Pharmacy Timisoara, Eftimie Murgu Square 2, 300041 Timisoara, Romania

**Keywords:** miRNA, laryngeal cancer, oncology, otolaryngology

## Abstract

Background and Objectives: Laryngeal cancer poses a significant clinical challenge, with late-stage diagnosis contributing to high morbidity and mortality. Circulating microRNAs (miRNAs) represent promising, minimally invasive biomarkers for earlier detection and improved therapeutic monitoring. This pilot study focused exclusively on miRNAs found to be upregulated in laryngeal carcinoma patients, aiming to elucidate their diagnostic and prognostic relevance. Methods: A total of 50 participants meeting standardized inclusion criteria were recruited from the ENT Clinic in Timișoara. Of these, 30 patients provided paired blood samples before and after treatment (surgical or non-surgical). Samples were pooled into three preoperative groups (P1, P2, P3) and three corresponding postoperative groups (C1, C2, C3). miRNAs were extracted from plasma and exosomes, and relative expression was measured by qPCR (Qiagen platform). Statistical analyses included Mann–Whitney U tests, receiver operating characteristic (ROC) curves, and logistic regression. Results: Seven miRNAs consistently exhibited significant upregulation preoperatively. Notably, hsa-miR-424-5p displayed a mean fold change of 4.59 (*p* = 0.0091) relative to postoperative samples, while hsa-miR-186-5p increased by 2.19-fold (*p* = 0.0030). hsa-miR-15b-5p also showed a substantial preoperative upregulation of 1.77-fold (*p* = 0.0057). In ROC analyses, hsa-miR-424-5p yielded an area under the curve (AUC) of 0.82 (95% CI 0.70–0.94), with 78% sensitivity and 80% specificity in distinguishing preoperative from postoperative status. Logistic regression indicated that elevated levels of hsa-miR-424-5p (OR = 1.56, 95% CI 1.10–2.20) and hsa-miR-186-5p (OR = 1.32, 95% CI 1.02–1.68) significantly predicted the preoperative disease state. Conclusions: These data underscore the potential of upregulated circulating miRNAs to serve as biomarkers for active laryngeal cancer and to monitor treatment response. Although preliminary, the findings encourage further research with larger cohorts and additional endpoints. With thorough validation, upregulated miRNAs could be integrated into clinical workflows, enhancing diagnostic precision, risk stratification, and postoperative surveillance in laryngeal cancer.

## 1. Introduction

Laryngeal cancer remains a formidable clinical problem, often diagnosed in advanced stages when treatment outcomes are less favorable [1,2]. Against this backdrop, the search for reliable, minimally invasive biomarkers has intensified [3,4]. In recent years, particular attention has been paid to microRNAs (miRNAs)—small, non-coding RNA molecules that regulate gene expression—due to their remarkable stability in circulation and strong correlation with tumor biology [5,6,7].

Upregulated miRNAs often reflect oncogenic processes, potentially driving proliferation, angiogenesis, or immune evasion [8,9,10]. Understanding these altered miRNAs can illuminate critical pathways that either maintain the malignant phenotype or facilitate disease progression. Emerging data indicate that circulating miRNAs can outperform some traditional blood-based markers in sensitivity and specificity, particularly when analyzed collectively in panels [11,12,13].

By concentrating on upregulated miRNAs, researchers may gain a more refined molecular signature that corresponds to active tumor states [14,15]. Identifying miRNA profiles before and after therapeutic intervention could greatly enhance patient monitoring, as previously demonstrated in studies of other types of cancer [16,17]. Moreover, by extracting miRNAs from both plasma and exosomal fractions, researchers can achieve a comprehensive snapshot of tumor-derived epigenetic signals [18,19]. Exosomes protect their cargo from enzymatic degradation, potentially reflecting active oncogenic processes more accurately [20,21]. Analyzing these fractions in tandem could yield insights into how tumor cells release and utilize miRNAs as functional mediators.

Ultimately, the successful identification of consistently upregulated miRNAs might enable clinicians to predict treatment response, stratify risk, and optimize follow-up schedules. Beyond diagnostics, these dysregulated miRNAs may also serve as targets for novel therapies. Our pilot findings, though preliminary, highlight the feasibility of such an approach and set the stage for larger-scale investigations aimed at integrating miRNA signatures into standard laryngeal cancer care.

## 2. Materials and Methods

### 2.1. Study Design and Participants

This pilot investigation enrolled 50 individuals with histopathologically confirmed laryngeal carcinoma, recruited from the ENT Clinic at the Clinical Municipal Hospital, affiliated with Victor Babes University of Medicine and Pharmacy in Timisoara. Patients were selected according to standardized criteria (DSM-IV-TR for laryngeal cancer), which included an age range of 18–85 years, and all provided informed consent. Those who had not undergone at least one year of post-therapeutic monitoring or who refused blood collection were excluded. Out of the 50 participants, a subset of 30 provided paired blood samples before and after therapeutic intervention (either surgical resection or non-surgical treatment). These 30 preoperative and 30 postoperative samples were further divided into three pools (each containing 10 patient samples) to generate broader expression profiles).

The study was conducted according to the guidelines of the Declaration of Helsinki and approved by the Ethics Committee of Victor Babes University of Medicine and Pharmacy, Timisoara, on 6 April 2023 (approval code I-9647). All participants provided informed consent, affirming their voluntary participation and understanding of the research purposes, procedures, and potential risks involved.

### 2.2. Sample Collection and Processing

Peripheral venous blood (2 × 3 mL tubes) was drawn using standardized vacutainer systems by experienced medical personnel to minimize complications such as hematoma or discomfort. Tubes were promptly transported on ice to the Discipline of Biochemistry department at Victor Babeș University of Medicine and Pharmacy, Timișoara (UMFVBT), where plasma separation was performed by centrifugation. Exosomes were isolated from a portion of each plasma sample using differential centrifugation and commercially available purification kits. Total RNA, inclusive of miRNAs, was extracted from both the free-circulating and exosomal fractions. Careful labeling and chain-of-custody procedures ensured sample integrity throughout the process. RNA extracts were stored at −80 °C until reverse transcription and qPCR analysis.

### 2.3. Laboratory Analysis of Circulating Markers

Total RNA was reverse-transcribed into cDNA using a miRNA-specific kit (Qiagen). Each pooled sample underwent quantitative real-time PCR (qPCR) using a high-throughput panel for human miRNAs, incorporating controls such as UniSP2, UniSP4, UniSP5, and an external spike-in (miR-39 cel) to validate amplification efficiency. UniSP3 served as an inter-plate calibrator. Expression data were captured as threshold cycle (Ct) values, which were normalized using multiple reference strategies (per Qiagen’s platform). Relative quantification relied on the 2^(−ΔCt) method to determine fold regulation. Here, a positive fold regulation indicated upregulation in preoperative samples relative to postoperative controls. Rigorous quality-control checks ensured consistency across plates, and any outlier data underwent additional review before inclusion in the final analysis.

Table 1 outlines the design used to generate average expression profiles from 30 preoperative and 30 postoperative blood samples. Each pool comprises 10 individual samples, providing a snapshot of the collective miRNA expression within that cohort. This pooling approach is particularly advantageous in pilot studies, where it facilitates a broad exploratory screen of numerous miRNAs while simultaneously conserving reagents and reducing technical variability.

By creating three distinct preoperative pools (P1, P2, P3) and three corresponding postoperative pools (C1, C2, C3), the study maximizes the likelihood of identifying consistently upregulated miRNAs associated with tumor presence. Each pool thus represents the mean biological state of 10 patients who share a similar clinical timeline: pre-treatment for the P groups and 2–4 weeks post-treatment for the C groups. Although pooling can mask individual patient variability, it offers a practical means of discerning clear trends in miRNA expression—particularly those implicated in oncogenesis or active disease states.

In this study, the decision was made to pool samples into pre- and post-treatment groups primarily to optimize resource usage for this pilot investigation and to capture broader trends in miRNA expression. Given the relatively small cohort and the exploratory nature of our research, pooling allowed us to perform an initial screening of multiple miRNAs without depleting reagents or inflating experimental costs. Nonetheless, we recognize that this strategy may dilute individual-level variability and could potentially obscure unique expression profiles pertinent to specific patient subgroups. To mitigate this concern, we retained and reviewed individual Ct values for any outliers to ensure major discrepancies were not overlooked. In future studies, we intend to expand our sample size and conduct more granular analyses to validate findings at the individual patient level, thereby strengthening the reliability and translational relevance of our miRNA data.

### 2.4. Statistical Analysis

We performed an a priori sample size calculation using G*Power (version 3.1, University of Düsseldorf, Düsseldorf, Germany) to ensure sufficient power for detecting a two-fold change in miRNA expression (equivalent to a Cohen’s d of approximately 0.8). Specifically, we set the significance level (α) at 0.05 and aimed for 80% power (1 − β = 0.80). Based on pilot data from a prior internal study indicating a standard deviation of 1.2 in normalized miRNA expression (ΔCt values), this calculation suggested that at least 28 participants were required in each arm (preoperative vs. postoperative). Anticipating a potential dropout rate of around 10%, we enrolled a total of 50 individuals, ensuring that 30 provided paired samples (pre- and post-treatment), which met the minimum threshold for robust statistical comparisons.

Data were analyzed using SPSS Statistics version 27 (IBM Corp., Armonk, NY, USA). Normality was assessed using the Shapiro–Wilk test. Because most miRNA distributions deviated from normality, non-parametric tests—chiefly the Mann–Whitney U test—were employed to compare preoperative vs. postoperative expression levels. Spearman’s rank correlation explored relationships among significantly upregulated miRNAs. Receiver operating characteristic (ROC) curve analyses were performed for selected miRNAs to evaluate their ability to discriminate between pre- and postoperative states. Sensitivity, specificity, and area under the curve (AUC) values were calculated. Logistic regression models further assessed the predictive power of top-performing miRNAs, with statistical significance set at *p* < 0.05. Potential confounders, including tumor stage, were considered where sample size permitted meaningful subgroup analyses.

## 3. Results

A total of 50 laryngeal cancer patients, predominantly male (76%) with a mean age of 60.8 years, participated in this study. Over half (56%) were current smokers, while 30% were former smokers. The most common comorbidity was hypertension (32%), followed by type 2 diabetes mellitus (20%) and COPD (18%). Stage III (30%) and IV (34%) disease accounted for nearly two-thirds of the cohort, and glottic tumors were the most frequent (60%). Primary treatment was non-surgical for the majority (60%), whereas 40% underwent surgical resection, as presented in Table 2.

Table 3 presents the most upregulated miRNAs by comparing preoperative samples to postoperative samples in patients with laryngeal cancer. The miRNA hsa-miR-424-5p exhibited a mean ΔCt of −2.8 preoperatively and −0.6 postoperatively, resulting in a fold change of 4.59 and a *p*-value of 0.0091. hsa-miR-186-5p showed mean ΔCt values of 0.19 preoperatively and 1.32 postoperatively, with a fold change of 2.19 and a *p*-value of 0.003.

Additionally, hsa-miR-15b-5p exhibited mean ΔCt values of −2.9 preoperatively and −3.72 postoperatively, a fold change of 1.77, and a *p*-value of 0.0057. hsa-miR-34a-5p exhibited mean ΔCt values of 6.3 preoperatively and 4.01 postoperatively, with a fold change of 1.54 and a *p*-value of 0.02. hsa-miR-93-3p showed mean ΔCt values of 1.8 preoperatively and 0.03 postoperatively, a fold change of 1.5, and a *p*-value of 0.04. hsa-miR-200c-3p presented mean ΔCt values of 7.25 preoperatively and 3.69 postoperatively, a fold change of 2, and a *p*-value of 0.023. Lastly, hsa-miR-133a-3p showed mean ΔCt values of 3.63 preoperatively and 1.89 postoperatively, a fold change of 1.92, and a *p*-value of 0.035.

**Figure 1:**
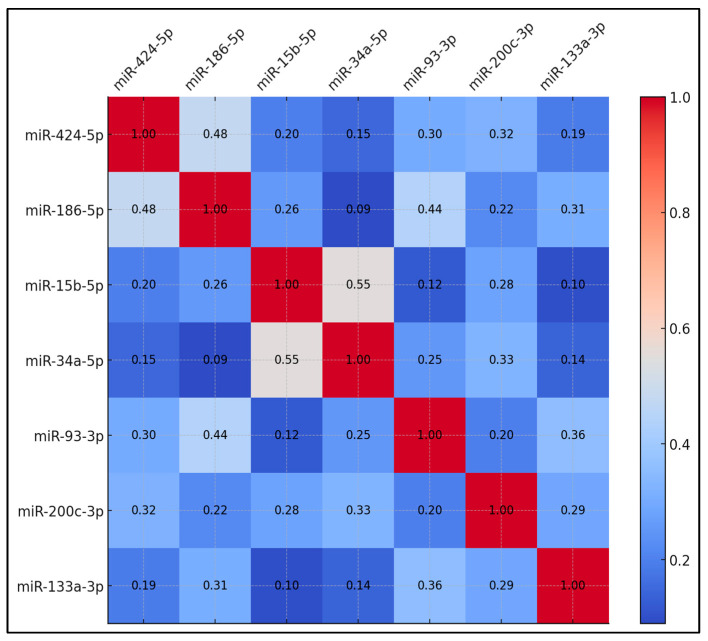
Correlation matrix for upregulated circulating miRNAs.

Table 4 displays the correlation coefficients among the upregulated miRNAs in preoperative samples. The correlation between miR-424-5p and miR-186-5p reached 0.48, which was statistically significant (*p* < 0.05). miR-15b-5p and miR-34a-5p showed a correlation coefficient of 0.55, significant at *p* < 0.01. Additionally, miR-186-5p and miR-93-3p showed a correlation of 0.44, which was significant (*p* < 0.05). Other miRNA pairs exhibited lower correlation coefficients. For example, miR-424-5p correlated with miR-15b-5p at 0.20, and miR-424-5p correlated with miR-34a-5p at 0.15. miR-93-3p and miR-133a-3p correlated at 0.36, while miR-200c-3p and miR-186-5p correlated at 0.22. The remaining miRNA pairs showed correlations ranging from 0.09 to 0.31 (Figure 1).

Table 5 presents the receiver operating characteristic (ROC) curve analysis for the upregulated miRNAs. miR-424-5p exhibited an area under the curve (AUC) of 0.82 with a 95% confidence interval (CI) of 0.70–0.94, a sensitivity of 78%, a specificity of 80%, and a *p*-value of 0.001. miR-186-5p showed an AUC of 0.79, a 95% CI of 0.66–0.92, a sensitivity of 74%, a specificity of 78%, and a *p*-value of 0.003.

Furthermore, miR-34a-5p had an AUC of 0.74, a 95% CI of 0.60–0.88, a sensitivity of 70%, a specificity of 70%, and a *p*-value of 0.02. miR-15b-5p exhibited an AUC of 0.77, a 95% CI of 0.62–0.92, a sensitivity of 72%, a specificity of 78%, and a *p*-value of 0.009. miR-200c-3p showed an AUC of 0.73, a 95% CI of 0.59–0.87, a sensitivity of 68%, a specificity of 72%, and a *p*-value of 0.023. miR-93-3p showed an AUC of 0.69, a 95% CI of 0.56–0.82, a sensitivity of 65%, a specificity of 70%, and a *p*-value of 0.04. Lastly, miR-133a-3p exhibited an AUC of 0.68, a 95% CI of 0.53–0.83, a sensitivity of 64%, a specificity of 66%, and a *p*-value of 0.048.

Table 6 details the logistic regression model used to predict preoperative status based on miR-424-5p and miR-186-5p. The odds ratio for miR-424-5p was 1.56 with a 95% confidence interval of 1.16–2.22 and a p-value of 0.010. The odds ratio for miR-186-5p was 1.32 with a 95% confidence interval of 1.07–1.68 and a *p*-value of 0.034.

## 4. Discussion

### 4.1. Analysis of Findings

This study set out to identify upregulated miRNAs in laryngeal cancer patients and to explore their clinical utility for diagnostics and treatment monitoring. The data show that several miRNAs, particularly miR-424-5p and miR-186-5p, exhibited substantial elevation preoperatively and trended toward normalization post-treatment. Correlation analyses further suggest that these miRNAs may belong to overlapping regulatory networks, though the exact mechanistic underpinnings remain to be elucidated. Compared to traditional biomarkers, miRNAs offer distinct advantages: stability in circulation, ease of quantification, and the potential to reflect ongoing tumor activity. Consequently, upregulated miRNAs might provide clinicians with a dynamic readout of disease status, allowing earlier detection of relapse or incomplete tumor eradication. Other findings underscored that these markers can discriminate between active and treated states with moderate to high sensitivity and specificity. While individual miRNAs rarely provide perfect diagnostic clarity, a panel-based approach could significantly boost accuracy. These insights strengthen the rationale for including miRNAs in multi-parameter predictive models that also incorporate imaging and histopathological data.

Table 7 provides an overview of the key biological pathways and processes potentially modulated by each upregulated miRNA, as identified through in silico analyses (e.g., target prediction databases and pathway enrichment tools). A recurring theme is the regulation of cell cycle and apoptosis-related pathways, evident for miR-424-5p [22], miR-15b-5p [23], and miR-34a-5p [24]. This aligns with well-established oncogenic mechanisms in which dysregulated control of proliferation and programmed cell death fosters malignant progression.

Notably, miR-186-5p appears to intersect with pathways involved in stress response and angiogenesis, such as MAPK and Notch [25]. Such pathways can be critical in tumor adaptation to microenvironmental pressures. Similarly, miR-93-3p [26] and miR-200c-3p are linked to epithelial–mesenchymal transition (EMT) and invasion, processes that facilitate metastasis. These connections hint that elevated miR-93-3p [27] or miR-200c-3p [28] might signal a transition to a more invasive phenotype, which is crucial knowledge for risk stratification and targeted therapies.

The predicted involvement of miR-133a-3p [29] in ERK/MAPK and Myc pathways underscores its possible regulatory influence on tumor growth and differentiation. Though functional validation remains essential, these in silico insights pave the way for targeted experimental work. Specifically, if certain miRNAs indeed govern key signaling nodes, they may become focal points for novel therapeutics or combination regimens. Overall, the pathways summarized here underscore how upregulated miRNAs could simultaneously serve as biomarkers of aggressive cancer biology and as potential levers for future precision oncology interventions.

In a similar manner, the study by Li Chen et al. [30] explored the impact of microRNA-141 (miR-141) on epithelial–mesenchymal transition (EMT) and lymph node metastasis in laryngeal cancer through the modulation of the Homeobox C6 (HOXC6)-dependent TGF-β signaling pathway. The researchers discovered that upregulation of miR-141 led to a downregulation of HOXC6 and subsequent inhibition of the TGF-β signaling pathway, which effectively repressed EMT and reduced the viability, migration, and invasion abilities of laryngeal cancer cells, as well as tumor growth in vivo. They further demonstrated that miR-141 upregulation notably curbed lymph node metastasis, suggesting a significant therapeutic potential in managing laryngeal cancer.

On the other hand, the study by Libing Guo et al. [31] investigated the expression levels of miRNA-145 and miRNA-218 in the serum of laryngeal cancer patients and their correlation with clinicopathological parameters and prognosis. Their findings indicated that lower serum levels of miRNA-145 and miRNA-218 were associated with poorer prognoses. Notably, patients in the high-expression group for these miRNAs demonstrated a median survival time of 30 months, compared to 26 months in the low-expression group, establishing these miRNAs as potential prognostic indicators. Both studies underscore the critical role of miRNAs in laryngeal cancer progression and prognosis, highlighting their utility in developing targeted therapeutic strategies and improving patient survival outcomes.

Moreover, the study by Fatma Ruya Tuncturk et al. explored the potential of microRNAs as transformation biomarkers in laryngeal lesions, distinguishing between benign, premalignant, and malignant stages [32]. They identified that certain miRNAs, such as Hs_miR-183_5p, Hs_miR-155_5p, and Hs_miR-106b_3p, were significantly upregulated in premalignant lesions compared to benign ones, with fold increases of 4.16, 2.72, and 3.01, respectively. As lesions progressed to malignancy, these upregulations continued, indicating a gradient of expression correlating with the severity of the lesion. Furthermore, miRNAs like Hs_miR-21_5p, Hs_miR-218_3p, and Hs_miR-210_3p exhibited substantial upregulation solely in the malignant stage compared to benign tissues, suggesting their specific association with fully transformed cancerous cells.

In parallel, the study by Pei Li et al. investigated the regulation and role of miR-1205 in LSCC, finding it notably downregulated in LSCC samples compared to adjacent non-tumorous tissues [33]. The authors further detailed how miR-1205 downregulation was linked with advanced clinical stage and poor prognosis, whereas its overexpression mitigated migration, growth, and invasion of LSCC cells. The study also highlighted a reciprocal regulatory mechanism between miR-1205 and E2F1, a transcription factor that was found to be inversely related to miR-1205 levels and associated with poorer clinical outcomes. Both studies underscore the nuanced roles of specific miRNAs in the progression from benign to malignant states and their potential utility in clinical diagnostics and prognostics, reflecting the complexity and promise of miRNA-based markers in cancer management.

Genetic polymorphisms in miRNA biogenesis and function can modulate circulating miRNA levels, influencing both their expression and regulatory capacities across individuals. Given that our pilot study did not analyze these genetic variations, the observed miRNA alterations may not be generalizable to populations with different polymorphic profiles. For example, if multiple participants share a common variant that impacts miRNA processing enzymes, the resulting upregulation or downregulation could be population specific. The absence of genotyping data therefore constrains our ability to interpret whether the detected miRNA shifts reflect inherent tumor biology or an interplay between genetics and disease state. Future investigations should incorporate genetic screening to ensure more robust conclusions on the role of miRNAs in laryngeal cancer and improve the translatability of findings across diverse cohorts [34,35,36].

The miRNAs highlighted in this study, while showing a clear association with laryngeal cancer, have not been examined here for their mechanistic roles in tumor biology. Elucidating how each miRNA modulates gene expression—whether by fostering proliferation, enabling metastasis, or instead acting as a gatekeeper against tumor progression—is essential for interpreting their clinical significance. Some may drive oncogenic pathways, whereas others could inhibit critical steps in tumorigenesis. By delineating these specific targets and pathways, future research can more precisely determine if such miRNAs might serve as viable therapeutic targets or molecular indicators of disease status.

Our findings indicate that these upregulated miRNAs show promise as potential biomarkers for detecting and monitoring laryngeal cancer, but the path from pilot data to routine clinical application remains multifaceted. Rather than supplanting established diagnostic methods, these miRNAs would likely augment existing tools—such as imaging and histopathology—providing an additional layer of molecular specificity. The next steps involve conducting larger-scale, multicenter trials to confirm our results, refining cut-off thresholds for diagnostic accuracy, and performing longitudinal monitoring to see how miRNA levels correlate with clinical outcomes over time. In parallel, targeted functional studies could verify whether modulating these miRNAs affects tumor behavior, thereby validating their mechanistic role in laryngeal cancer progression.

A longitudinal approach tracking miRNA levels at multiple time points would likely yield more nuanced insights into their roles in disease progression and therapy response. Such temporal data could capture dynamic fluctuations, as individual patients may exhibit unique expression patterns in response to different treatments. By examining miRNA shifts throughout the entire course of the disease, researchers can better understand whether these biomarkers reflect ongoing tumor biology or merely static snapshots of disease state. Deepening the exploration of the molecular pathways that these miRNAs regulate would significantly enhance our insight into their roles in laryngeal cancer. Pathway enrichment analyses, coupled with knockdown and overexpression experiments, could pinpoint the specific oncogenic or tumor-suppressive mechanisms of each miRNA.

### 4.2. Study Limitations

Several limitations should be addressed when interpreting these results. First, the pilot design and modest sample size restrict the generalizability of our findings. Pooling patient samples, while cost-effective and practical for an initial screening, can obscure individual-level variability that may be clinically meaningful. Second, the study examined only a single postoperative time window (2–4 weeks), potentially missing ongoing fluctuations in miRNA expression related to wound healing, inflammation, or longer-term tumor recurrence. Third, despite analyzing both free-circulating and exosomal fractions, we did not explore other epigenetic modifications (e.g., DNA methylation, long non-coding RNAs), which could offer a more complete molecular picture. Fourth, our in silico pathway predictions, though informative, are hypothetical until experimentally validated. Moreover, the study assesses miRNA levels at a single postoperative time point (2–4 weeks), which may not capture dynamic changes in expression over time. Finally, factors such as smoking status, comorbidities, or genetic polymorphisms in miRNA biogenesis might confound expression patterns but were not deeply interrogated here. Larger, longitudinal, and multicenter trials are necessary to confirm the diagnostic and prognostic potential of these upregulated miRNAs.

## 5. Conclusions

In conclusion, this cross-sectional pilot study underscores the feasibility and potential clinical relevance of focusing on upregulated miRNAs in laryngeal cancer. Our data pinpoint miR-424-5p, miR-186-5p, and others that exhibit significant overexpression prior to treatment, followed by decreased levels post-intervention. These changes suggest a strong link between miRNA expression patterns and active tumor biology. Through correlation, logistic regression, and ROC analyses, we demonstrate that upregulated miRNAs can distinguish preoperative from postoperative states with promising sensitivity and specificity.

Beyond diagnostics, these findings carry implications for translational medicine. Upregulated miRNAs may serve dual roles: first, as accessible blood-based markers for tracking disease status and therapeutic efficacy; and second, as potential oncomiRs implicated in proliferative, angiogenic, and metastatic pathways. Although further validation is necessary, this pilot work lays the groundwork for more extensive research to integrate miRNA panels into routine clinical workflows. Ultimately, harnessing these biomarkers could improve patient outcomes by enabling earlier detection, refined prognostication, and personalized treatment strategies for laryngeal cancer.

## Figures and Tables

**Table 1:** Overview of pooled pre-treatment and post-treatment samples.

Group	Sample Source	Number of Pooled Samples	Time Point
P1	Pre-Treatment (Plasma)	10	Before Intervention
P2	Pre-Treatment (Plasma)	10	Before Intervention
P3	Pre-Treatment (Plasma)	10	Before Intervention
C1	Post-Treatment (Plasma)	10	2–4 Weeks After
C2	Post-Treatment (Plasma)	10	2–4 Weeks After
C3	Post-Treatment (Plasma)	10	2–4 Weeks After

**Table 2:** Background characteristics.

Characteristic	Total (N = 50)
Age, years	Mean (SD): 60.8 (±9.3)
	Range: 45–80
Sex	Male: 38 (76%)
	Female: 12 (24%)
Body Mass Index (BMI), kg/m²	Mean (SD): 26.1 (±3.7)
Smoking Status	Current: 28 (56%)
	Former: 15 (30%)
	Never: 7 (14%)
Alcohol Consumption	Regular (≥3 drinks/week): 20 (40%)
	Occasional (<3 drinks/week): 15 (30%)
	None: 15 (30%)
Tumor Stage	I: 8 (16%)
	II: 10 (20%)
	III: 15 (30%)
	IV: 17 (34%)
Tumor Subsite	Glottic: 30 (60%)
	Supraglottic: 15 (30%)
	Subglottic: 5 (10%)
Primary Treatment	Surgical Resection: 20 (40%)
	Non-surgical (RT ± CT): 30 (60%)
Comorbidities	
Hypertension	16 (32%)
Type 2 Diabetes Mellitus	10 (20%)
Chronic Obstructive Pulmonary Disease	9 (18%)
Cardiovascular Disease	6 (12%)
Chronic Kidney Disease	3 (6%)
Multiple Comorbidities (≥2)	12 (24%)

**Table 3:** Most upregulated miRNAs in preoperative vs. postoperative samples.

miRNA	Mean ΔCt Pre-Op	Mean ΔCt Post-Op	Fold Change (Pre vs. Post)	*p*-Value
hsa-miR-424-5p	−2.8	−0.6	4.59	0.0091
hsa-miR-186-5p	0.19	1.32	2.19	0.003
hsa-miR-15b-5p	−2.9	−3.72	1.77	0.0057
hsa-miR-34a-5p	6.3	4.01	1.54	0.02
hsa-miR-93-3p	1.8	0.03	1.5	0.04
hsa-miR-200c-3p	7.25	3.69	2	0.023
hsa-miR-133a-3p	3.63	1.89	1.92	0.035

**Table 4:** Correlation matrix among upregulated miRNAs (preoperative samples).

miRNA	miR-424-5p	miR-186-5p	miR-15b-5p	miR-34a-5p	miR-93-3p	miR-200c-3p	miR-133a-3p
miR-424-5p	1	0.48 *	0.2	0.15	0.3	0.32	0.19
miR-186-5p	0.48 *	1	0.26	0.09	0.44 *	0.22	0.31
miR-15b-5p	0.2	0.26	1	0.55 **	0.12	0.28	0.1
miR-34a-5p	0.15	0.09	0.55 **	1	0.25	0.33	0.14
miR-93-3p	0.3	0.44 *	0.12	0.25	1	0.2	0.36
miR-200c-3p	0.32	0.22	0.28	0.33	0.2	1	0.29
miR-133a-3p	0.19	0.31	0.1	0.14	0.36	0.29	1

*—Statistically significant (*p* < 0.05); **—Statistically significant (*p* < 0.01).

**Table 5:** ROC curve analysis for upregulated miRNAs.

miRNA	AUC	95% CI	Sensitivity (%)	Specificity (%)	*p*-Value
miR-424-5p	0.82	0.70–0.94	78	80	0.001
miR-186-5p	0.79	0.66–0.92	74	78	0.003
miR-34a-5p	0.74	0.60–0.88	70	70	0.02
miR-15b-5p	0.77	0.62–0.92	72	78	0.009
miR-200c-3p	0.73	0.59–0.87	68	72	0.023
miR-93-3p	0.69	0.56–0.82	65	70	0.04
miR-133a-3p	0.68	0.53–0.83	64	66	0.048

**Table 6:** Logistic regression model predicting preoperative status using miR-424-5p and miR-186-5p.

Variable	Odds Ratio (95% CI)	*p*-Value
miR-424-5p	1.56 (1.16–2.22)	0.010
miR-186-5p	1.32 (1.07–1.68)	0.034
Constant	–	0.001

**Table 7:** Potential roles and clinical significance of upregulated circulating miRNAs.

miRNA	Predicted Target Pathways	Key Biological Processes	Potential Clinical Relevance
miR-424-5p	Cell Cycle, PI3K-Akt, TGF-β Signaling	Proliferation, Apoptosis	Therapeutic targeting of cell cycle mediators
miR-186-5p	MAPK, Notch, DNA Damage Response	Stress Response, Angiogenesis	Biomarker for monitoring therapeutic response
miR-15b-5p	Apoptotic Pathways, p53 Signaling	Cell Death Regulation	Potential synergy with chemotherapeutic agents
miR-34a-5p	p53 Pathway, Cell Cycle Arrest	Senescence, DNA Repair	Marker for advanced disease; tumor suppression
miR-93-3p	E2F Transcription, EMT	Proliferation, Invasion	Predictor of metastatic potential
miR-200c-3p	EMT, Wnt/β-catenin Signaling	Migration, Epithelial Differentiation	Prognostic indicator for aggressive tumors
miR-133a-3p	ERK/MAPK, Myc Pathway	Growth Regulation, Differentiation	Possible role in halting tumor progression

## Data Availability

The data presented in this study are available on request from the corresponding author.
